# Evaluation of three mechanisms of action (SGLT2 inhibitors, GLP-1 receptor agonists, and sulfonylureas) in treating type 2 diabetes with heart failure: a systematic review and network meta-analysis of RCTs

**DOI:** 10.3389/fendo.2025.1562815

**Published:** 2025-06-10

**Authors:** Huize Gao, Qian Wei, Anqi Zou, Keying Yu, Da Song, Jian Li, Huize Han, Aidong Liu

**Affiliations:** ^1^ Changchun University of Chinese Medicine, Changchun, Jilin, China; ^2^ The Third Affiliated Hospital of Changchun University of Chinese Medicine, Changchun, Jilin, China; ^3^ The Affiliated Hospital of Changchun University of Chinese Medicine, Changchun, Jilin, China

**Keywords:** type 2 diabetes with heart failure, SGLT2 inhibitors, GLP-1 receptor agonists, efficacy and safety, network meta-analysis

## Abstract

**Objective:**

We aimed to evaluate and compare the efficacy and safety of three antidiabetic drug classes—SGLT2 inhibitors, GLP-1 receptor agonists, and sulfonylureas—in patients with type 2 diabetes mellitus (T2DM) complicated by heart failure (HF). We focused on their differential effects on both cardiovascular outcomes (e.g., heart failure biomarkers and cardiac function) and metabolic outcomes (e.g., glycemic control and body weight), aiming to determine whether the newer agents offer superior cardiometabolic benefits. A network meta-analysis was conducted to integrate available evidence and compare all interventions simultaneously.

**Methods:**

A comprehensive literature search was performed in PubMed, EMBASE, and the Cochrane Library. encompassing all available records up to December 10, 2024. Fourteen RCTs were included. A Bayesian network meta-analysis was utilized to integrate direct and indirect evidence, facilitating a comparative ranking of various SGLT2 inhibitors—canagliflozin (CANA), ipragliflozin (IPRA), empagliflozin (EMPA), remogliflozin (REMO), licogliflozin (LICO), and dapagliflozin (DAPA)—as well as one GLP-1 receptor agonist—semaglutide (SEMA)—and a sulfonylurea—glimepiride (GLIM)—with respect to their efficacy and safety profiles.

**Results:**

SEMA (SMD = –0.22, 95% CI: –1.31 to 0.87) demonstrated the most favorable outcome in reducing BNP levels. LICO (SMD = –0.91, 95% CI: –1.76 to –0.06) ranked highest for body weight reduction, indicating the greatest impact. GLIM (SMD = –0.64, 95% CI: –1.12 to –0.17) showed the strongest effect on lowering HbA1c, while DAPA (SMD = 0.34, 95% CI: –0.97 to 1.65) was the top-ranked agent for improving LVEF. Safety analysis indicated that LICO and IPRA had the lowest incidence of adverse events. GLIM was associated with an increased risk of hypoglycemia, whereas DAPA was linked to a higher risk of urinary tract infections.

**Conclusion:**

SEMA significantly improves both metabolic control and BNP levels, making it suitable for patients requiring comprehensive management of metabolic abnormalities and heart failure. LICO offers a distinct advantage in weight management, particularly benefiting individuals with obesity. DAPA demonstrates notable efficacy in optimizing HbA1c and LVEF, making it a preferred option for patients needing more intensive cardiac support. Despite its moderate efficacy, GLIM remains a viable choice for certain patients due to its favorable safety profile and cost-effectiveness. Collectively, these findings provide essential evidence-based insights to guide individualized therapeutic strategies in type 2 diabetes complicated by heart failure.

## 1 Introduction

Heart failure (HF) is among the most prevalent and fatal cardiovascular diseases worldwide, affecting an estimated 64.3 million individuals globally (1). The five-year mortality rate for HF patients can reach up to 50%, underscoring its significance as a primary clinical concern (2). Diabetes mellitus is a prevalent comorbidity in heart failure, affecting approximately 40% of patients with heart failure with reduced ejection fraction (HFrEF) and 45% of those with heart failure with preserved ejection fraction (HFpEF) (3). This association is largely due to hyperglycemia-induced metabolic disturbances such as oxidative stress, endothelial dysfunction, and advanced glycation end product accumulation, all of which contribute to cardiomyocyte injury and ventricular remodeling (4). Consequently, managing diabetes in patients with heart failure presents a complex and critical challenge, necessitating therapeutic agents that can simultaneously enhance both cardiac function and metabolic status. Current pharmacotherapies for heart failure include angiotensin-converting enzyme inhibitors (ACEIs), angiotensin II type 1 receptor blockers (ARBs), β-blockers, mineralocorticoid receptor antagonists (MRAs), and angiotensin receptor-neprilysin inhibitors (ARNIs) such as sacubitril/valsartan (5). However, these standard therapies present limitations for heart failure patients with concurrent diabetes. For instance, conventional hypoglycemic agents, including sulfonylureas, have been linked to an elevated risk of heart failure in certain studies (6). Therefore, there is a need to develop comprehensive therapeutic strategies with enhanced cardiovascular protective effects. Novel agents, such as sodium–glucose cotransporter-2 (SGLT2) inhibitors and glucagon-like peptide-1 (GLP-1) receptor agonists, are considered to provide potential benefits for patients with heart failure due to their unique mechanisms of action (7). SGLT2 inhibitors lower blood glucose levels and reduce blood volume by inhibiting the reabsorption of glucose and sodium in the renal tubules. Studies have demonstrated that they can significantly decrease hospitalization rates for heart failure and all-cause mortality. In addition, they improve myocardial energy metabolism, enhance diuresis and natriuresis, and exhibit anti-inflammatory and anti-fibrotic effects, collectively reducing cardiac preload and afterload (8). GLP-1 receptor agonists, including liraglutide and semaglutide, regulate blood glucose levels by enhancing insulin secretion, suppressing glucagon release, and delaying gastric emptying (9, 10). Furthermore, they provide additional cardiometabolic benefits, including reductions in body weight and improvements in blood pressure and lipid profiles. Emerging evidence also supports their antioxidative and anti-inflammatory effects on the vascular endothelium, which may contribute to attenuating atherosclerosis and improving cardiac outcomes (4).

Despite existing studies demonstrating some benefits of SGLT2 inhibitors and GLP-1 receptor agonists in patients with heart failure, most randomized controlled trials (RCTs) have been limited to comparisons of single or a few medications, lacking comprehensive evaluations across multiple drug classes.

To address this evidence gap, we performed a Bayesian network meta-analysis to compare the efficacy and safety of SGLT2 inhibitors, GLP-1 receptor agonists, and sulfonylureas in patients with T2DM and HF. This method allows for the integration of both direct and indirect comparisons across multiple therapies, and provides probabilistic rankings that are particularly valuable for clinical decision-making.

We hypothesized that SGLT2 inhibitors and GLP-1 receptor agonists would demonstrate superior cardiometabolic outcomes compared to sulfonylureas in patients with T2DM and HF.

## 2 Materials and methods

This network meta-analysis (NMA) was conducted in accordance with the Preferred Reporting Items for Systematic Reviews and Meta-Analyses extension statement for network meta-analyses (**Supplementary Table 1**). Given the limited availability of direct head-to-head randomized controlled trials (RCTs) comparing different drug classes in patients with type 2 diabetes mellitus (T2DM) and heart failure (HF),a Bayesian network meta-analysis framework was adopted to integrate both direct and indirect evidence across a connected network of interventions. This method enables the estimation of relative treatment effects and treatment rankings even when direct comparisons are sparse or unavailable. Compared to conventional pairwise meta-analysis, the Bayesian approach offers greater flexibility in modeling multi-arm comparisons, incorporates prior distributions, and provides probabilistic statements regarding the likelihood of each treatment being optimal. This analytic strategy is particularly suitable for decision-making involving multiple competing treatments, such as the three drug classes under investigation. Accordingly, we applied this approach to estimate and compare the relative efficacy and safety of SGLT2 inhibitors, GLP-1 receptor agonists, and sulfonylureas. To ensure transparency, reliability, and novelty, the study protocol was registered in the Prospective Register of Systematic Reviews (CRD42025630552).

### 2.1 Data sources and search strategy

A systematic literature search was performed in PubMed, EMBASE, and the Cochrane Library. The search terms included “heart failure,” “cardiac failure,” “myocardial failure,” “heart decompensation,” “randomized clinical trial,” “RCT,” “type 2 diabetes mellitus,” “T2DM,” “non-insulin dependent diabetes,” “type 2 diabetes,” “maturity onset diabetes,” “noninsulin dependent diabetes mellitus,” “adult-onset diabetes mellitus,” and “ketosis-resistant diabetes mellitus.” The search period spanned from the inception of each database up to December 10, 2024. The search strategy employed a combination of free-text terms and controlled vocabulary, with no restrictions on language.

### 2.2 Selection criteria

Inclusion Criteria

(1)RCTs: Studies involving adult patients with a confirmed diagnosis of T2DM and concurrent HF.(2)Interventions: RCTs evaluating monotherapy or combination therapy with SGLT2 inhibitors, GLP-1 receptor agonists, or sulfonylureas.(3)Comparators: RCTs comparing the specified interventions with placebo, SOC, or other pharmacological treatments.(4)Outcome Measures: RCTs reporting at least one of the following outcomes, BNP Levels: Changes in brain natriuretic peptide (BNP) concentrations or other related biomarkers. Body Weight: Alterations in body weight or weight-related metrics. Glycated Hemoglobin (HbA1c): Changes in HbA1c levels. Left Ventricular Ejection Fraction (LVEF): Modifications in LVEF. Common Adverse Events: Incidence of mild to moderate adverse events, including but not limited to headache, nausea, diarrhea, and hypoglycemia.

Exclusion Criteria:

(1)Non-Randomized Studies: Observational studies, retrospective studies, and other non-RCT designs.(2)Multiple Phases: RCTs investigating different stages of the same patient cohort.(3)Ineligible Interventions: Studies involving medications outside the specified classes (i.e., non-SGLT2 inhibitors, non-GLP-1 receptor agonists, or non-sulfonylurea drugs).(4)Unclear Outcomes: RCTs that do not clearly define or report the specified outcome measures.(5)Non-Original Research: Reviews, case reports, and other studies that do not present original data. This structured approach ensures the inclusion of high-quality evidence relevant to the comparative efficacy and safety of SGLT2 inhibitors, GLP-1 receptor agonists, and sulfonylureas in managing patients with T2DM and HF, while excluding studies that do not meet the rigorous standards necessary for a robust network meta-analysis.

### 2.3 Data extraction and quality assessment

Three researchers independently extracted data from the RCTs in accordance with the Preferred Reporting Items for Systematic Reviews and Meta-Analyses (PRISMA) guidelines. In instances of discrepancies, a fourth author was consulted to achieve consensus. The extracted data from each study encompassed the first author’s name, sample size, year of publication, randomization method, median age in both the intervention and control groups, gender distribution within each group, and the treatment regimens administered to the intervention and control cohorts.

The quality of the included RCTs was evaluated using the Cochrane Risk of Bias Tool (Version 2.0). This instrument assesses five domains:(1)Risk of bias arising from the randomization process (2)Risk of bias due to deviations from intended interventions (3)Risk of bias from missing outcome data (4)Risk of bias in the measurement of the outcome (5)Risk of bias in the selection of the reported result.

Each RCT was assigned a risk level of low, high, or “some concerns” for each domain. This thorough quality assessment ensures the reliability and validity of the findings obtained from the network meta-analysis.

### 2.4 Statistical analysis

Bayesian network meta-analyses were performed using Stata 17.0. For dichotomous outcomes, odds ratios (ORs) were utilized as the effect size metric, whereas continuous outcomes were expressed as mean differences (MDs) with corresponding 95% confidence intervals (CIs). When continuous variables were assessed using different units, standardized mean differences (SMDs) were calculated to reduce heterogeneity.

In constructing the network evidence diagrams, the size of each node represented the sample size of the respective intervention, and the thickness of the connecting lines indicated the number of RCTs comparing the two interventions. For networks exhibiting an open-loop structure, a consistency model was applied. In contrast, for closed-loop structures, inconsistency tests were conducted to evaluate the coherence of outcome measures. A p-value greater than 0.05 suggested satisfactory consistency between direct and indirect evidence, thereby justifying the use of a consistency model.

Although the overall inconsistency tests yielded p ≥ 0.05, suggesting acceptable coherence, subgroup analyses and meta-regression were not feasible due to the limited number of included studies with stratified subgroups (e.g., HFrEF *vs*. HFpEF).

This limitation was acknowledged, and caution was taken when interpreting potential heterogeneity across treatment classes.

Subsequently, cumulative probability ranking plots were generated based on the surface under the cumulative ranking (SUCRA) values to determine the most efficacious treatment regimen. For networks with closed-loop structures, loop-specific inconsistency tests were implemented to assess the consistency within each loop. A 95% confidence interval for the inconsistency factor that included zero indicated good concordance between direct and indirect evidence.

Finally, comparison-adjusted funnel plots were employed to assess potential publication bias and the presence of small-study effects. Visual inspection and symmetry of funnel plots were used to evaluate the likelihood of reporting bias.

## 3 Results

### 3.1 Systematic review and characteristics of the included studies

**Figure 1:**
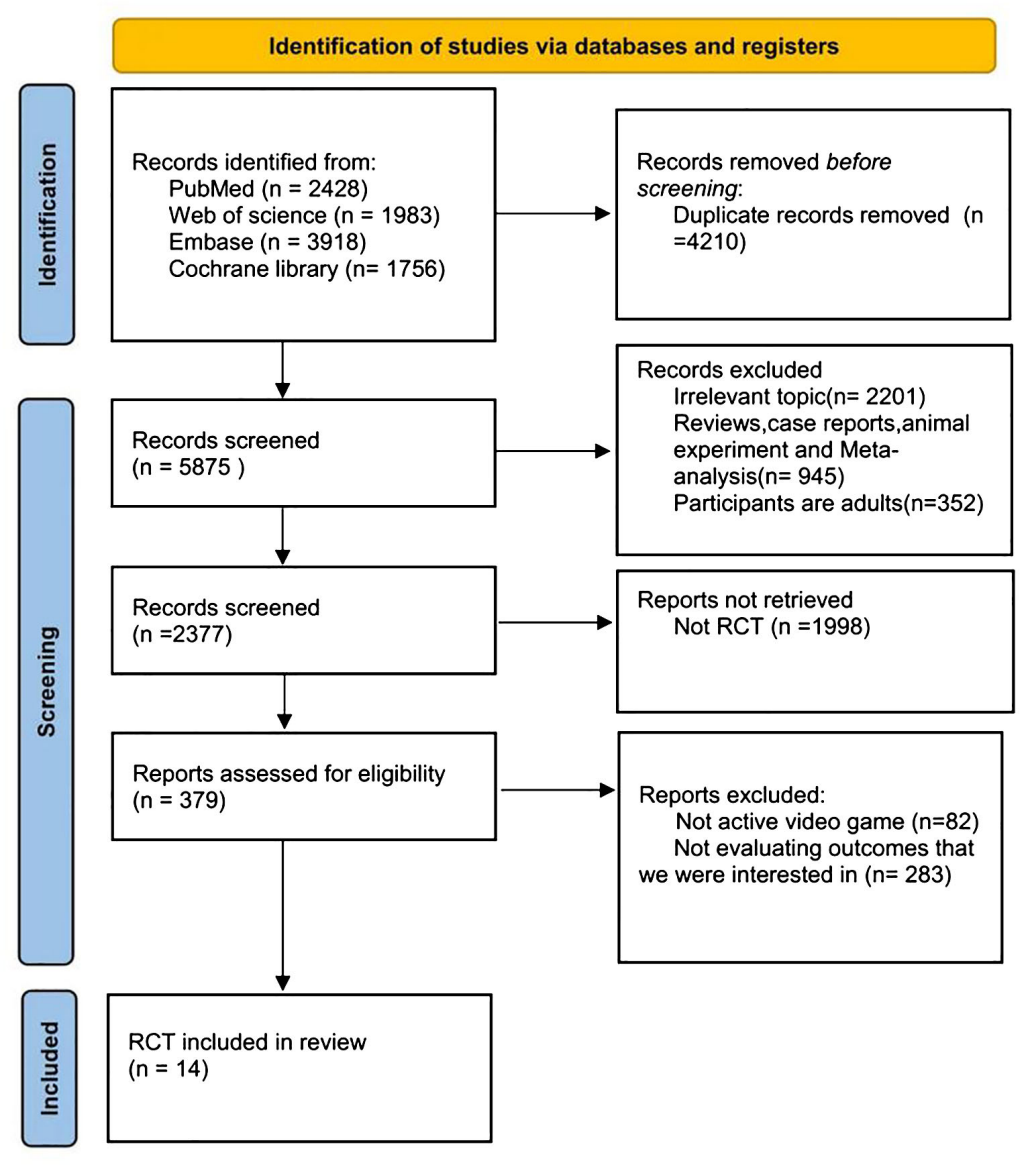
PRISMA (Preferred Reporting Items for Systematic Reviews and Meta-Analyses) flow diagram for selection and inclusion of the studies via databases. Latest search date: Dec, 2024.

The initial literature search yielded a total of 5,875 records from the databases. After screening abstracts to eliminate duplicates and irrelevant studies, 379 articles were deemed eligible for full-text review. Ultimately, 14 (11–24) studies met our inclusion criteria (**Figure 1**).

**Figure 2:**
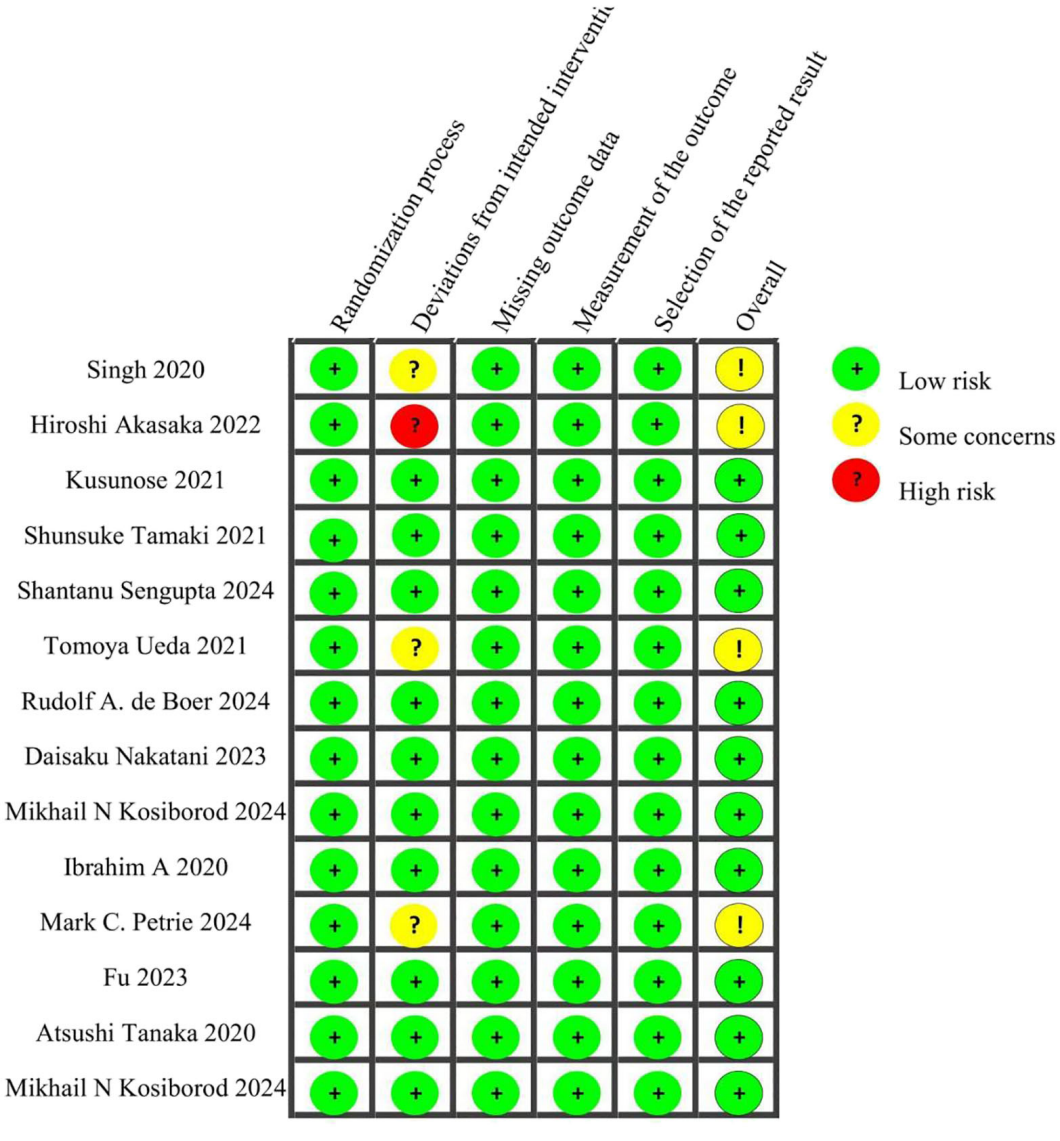
Detailed quality assessment results.

This analysis included 6,931 patients who were randomly assigned to receive one of the following treatment regimens: canagliflozin (CANA), ipragliflozin (IPRA), empagliflozin (EMPA), remogliflozin (REMO), licogliflozin (LICO), semaglutide (SEMA), dapagliflozin (DAPA), or standard of care (SOC). The control groups comprised glimepiride (GLIM) and other conventional treatments. Detailed information for all included studies is provided in **Table 1**. Quality assessment was conducted using the Cochrane Risk of Bias Tool (ROB 2.0). Among the 14 studies evaluated, 10 were assessed as having a low overall risk of bias, while 4 were categorized as having “some concerns.” Detailed quality assessment results are presented in **Figure 2**.

**Table 1:** Baseline characteristics of studies included in the network meta-analysis.

First Author	Sample Size	Year	Randomization	Median age(Experimental Group)	Median age(Control Group)	Male/Famale(Experimental Group)	Male/Famale(Control Group)	Intervention Arm	Control Arm
Kazuki Shiina	113/120	2020	1:1	68.3 ± 9.8	68.9 ± 10.4	78/35	84/36	Canagliflozin	Glimepiride
Kenya Kusunose	113/120	2021	1:1	69 ± 9	69 ± 9	88/25	86/34	Canagliflozin	Glimepiride
Shunsuke Tamaki	30/29	2021	1:1	80	82	18/12	18/11	Empagliflozin	Conventional glucose- lowering therapy
Shantanu Sengupta	125/125	2024	1:1					Remogliflozine	Empagliflozine
Ueda T	42/40	2021	1:1	76.5	75.9	28/14	27/13	Canagliflozin	Standard Diabetic Therapy
Rudolf A. de Boer	30/30/33	2024	1:1	66	68.5	28/2	24/6	Licogliflozin	Empagliflozin
			1:1		71		26/7		Placebo
Mikhail N Kosiborod	310/306	2024	1:1	64	65	195/115	186/120	Semaglutide	Placebo
M.N. Kosiborod	310/306	2024	1:1	69	70	128/182	145/161	semaglutide	Placebo
Ibrahim A	50/50	2020	1:1	62.02 ± 8.8	60.64 ± 9.9	28/22	26/24	Dapagliflozin, furosemide, and other convent-ional anti-HF treatments.	Insulin for blood glucose control, furosemide, and other conventional anti-HF treatments
Mark C. Petrie	573/572	2024	1:1	70	72	192/192	215/163	Semaglutide	Placebo
Fu	30/30	2023	1:1	70.7	70.4	9/21	8/22	Dapagliflozin	Placebo
Daisaku Nakatani	109/117	2023	1:1	68.6	69.1	85/24	85/32	Canagliflozin	Glimepiride
Singh	28/28	2020	1:1	67.1	67.1	19/9	19/9	Dapagliflozin	Placebo

### 3.2 Network meta-analyses

#### 3.2.1 BNP outcome

**Figure 3:**
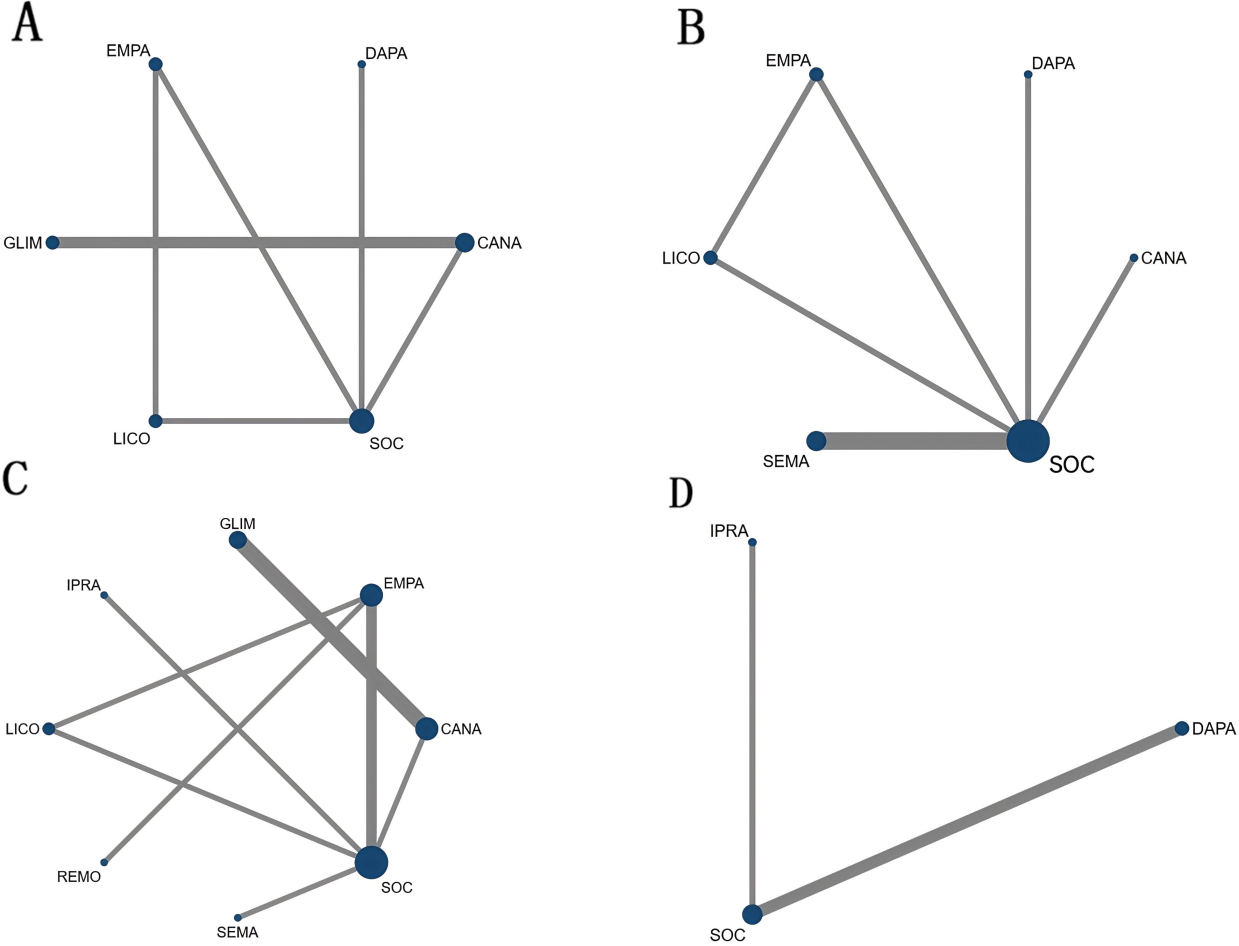
Network diagrams illustrating different drug interventions for the included subjects: **(A)** BNP network diagram. **(B)** Body weight network diagram.**(C)** HbA1c network diagram.**(D)** LVEF network diagram.

Nine studies reported changes in BNP levels, encompassing eight different treatment modalities within the network meta-analysis. The network diagram illustrating the various drug interventions for the included subjects is presented in **Figure 3A**. The BNP network formed a closed loop. An inconsistency test of the overall network yielded a p-value ≥ 0.05, indicating no significant inconsistency; therefore, a consistency model was utilized for the analysis. Loop-specific inconsistency tests revealed that the 95% confidence intervals included zero and that the inconsistency factors were minimal, suggesting good homogeneity. Ultimately, both direct and indirect comparisons demonstrated consistent results.

**Figure 4:**
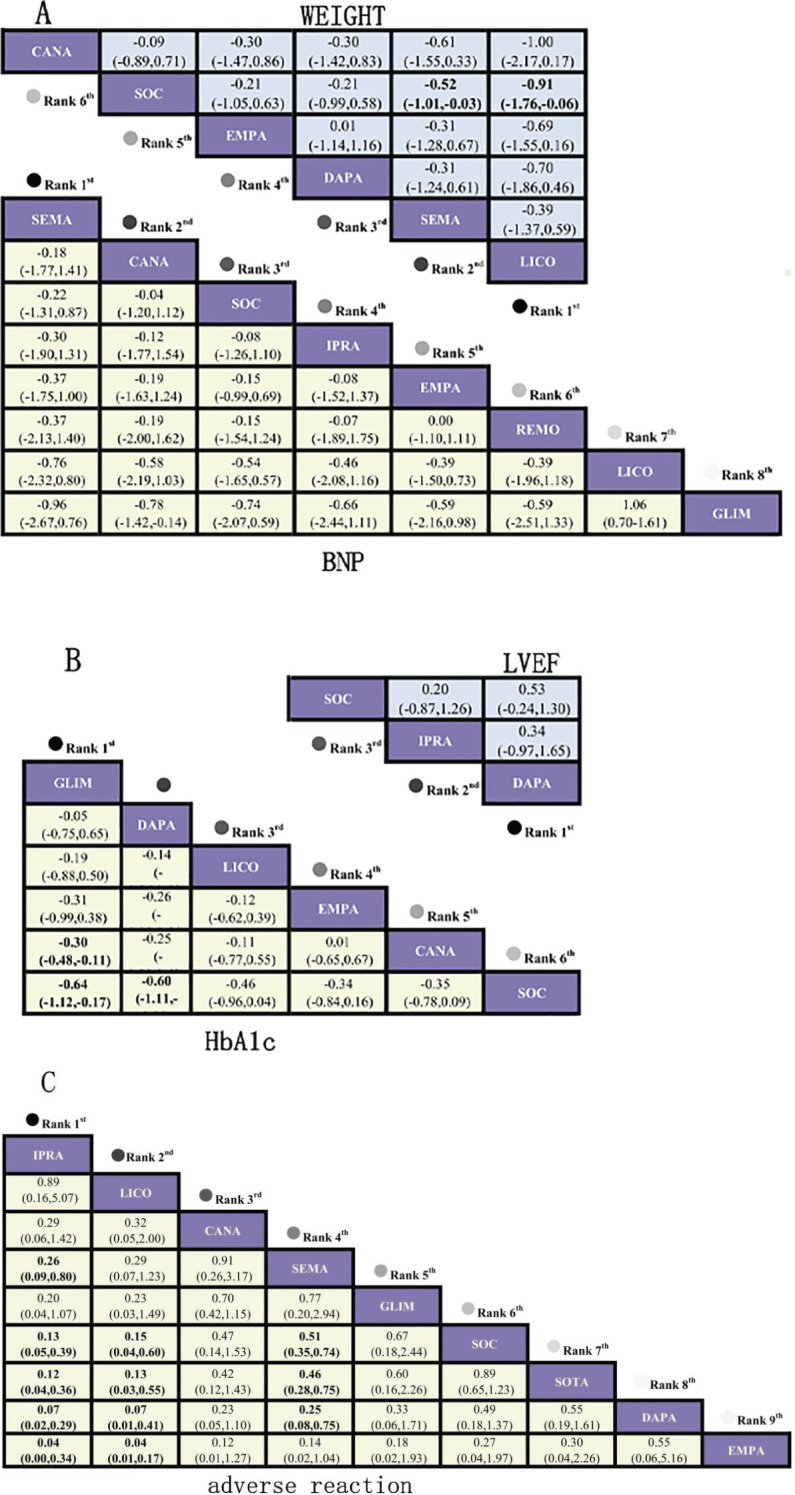
Rankograms illustrating comparative efficacy and safety of different medications across various outcome measures based on Bayesian network meta-analysis. **(A)** SMD and 95% CI for BNP (yellow lower triangle region) and body weight (blue upper triangle region). **(B)** SMD and 95% CI for HbA1c and LVEF. An SMD < 0.00 indicates a better survival benefit. **(C)** OR and 95% CI for Adverse Drug Reactions (ADRs).Note: SMD < 0.00 signifies a more favorable outcome in terms of survival benefits. OR < 1.00 signifies a reduced risk of adverse drug reactions, indicating better safety profiles.

SEMA (SMD = –0.22, 95% CI: –1.31 to 0.87) and CANA (SMD = –0.04, 95% CI: –1.20 to 1.12) showed some efficacy in improving BNP levels compared to SOC, although these differences were not statistically significant. CANA exhibited a slight advantage over SOC; however, this difference did not reach statistical significance. GLIM (SMD = 0.74, 95% CI: –0.59 to 2.07) did not demonstrate any benefit compared to SOC. Details are provided in **Figures 3A**, **4A**.

#### 3.2.2 Permission to reuse and copyright

Six studies reported changes in body weight, encompassing six distinct treatment modalities within the network meta-analysis. The network diagram illustrating the various drug interventions among the included subjects is presented in **Figure 3B**. The body weight network formed a closed loop. An inconsistency test of the overall network yielded a p-value ≥ 0.05, indicating no significant inconsistency; therefore, a consistency model was employed for the analysis. Loop-specific inconsistency tests revealed that the 95% confidence intervals included zero and that the inconsistency factors were minimal, suggesting good homogeneity. Consequently, both direct and indirect comparisons demonstrated consistent results.

LICO (SMD = –0.91, 95% CI: –1.76 to –0.06) and SEMA (SMD = –0.52, 95% CI: –1.01 to –0.03) showed efficacy in reducing body weight compared to SOC, with LICO reaching statistical significance. CANA (SMD = 0.09, 95% CI: –0.71 to 0.89) did not demonstrate any significant advantage over SOC in terms of body weight reduction.

#### 3.2.3 Glycated hemoglobin outcome 

Five studies reported changes in HbA1c levels, encompassing six distinct treatment modalities within the network meta-analysis. The network diagram illustrating the various drug interventions among the included subjects is presented in **Figure 3C**. The HbA1c network formed a closed loop. An inconsistency test of the overall network yielded a p-value ≥ 0.05, indicating no significant inconsistency; therefore, a consistency model was employed for the analysis. Loop-specific inconsistency tests revealed that the 95% confidence intervals included zero and that the inconsistency factors were minimal, suggesting good homogeneity. Consequently, both direct and indirect comparisons demonstrated consistent results.

GLIM (SMD = –0.64, 95% CI: –1.12 to –0.17) and DAPA (SMD = –0.60, 95% CI: –1.11 to –0.08) showed significant advantages in improving HbA1c levels compared to SOC. Additionally, GLIM (SMD = –0.30, 95% CI: –0.48 to –0.11) also demonstrated a significant advantage over CANA. Details are provided in **Figures 3C**, **4B**.

#### 3.2.4 Left ventricular ejection fraction outcome

Three studies reported changes in left ventricular ejection fraction (LVEF) levels, encompassing three distinct treatment modalities within the network meta-analysis. The network diagram illustrating the various drug interventions among the included studies is presented in **Figure 3D**. The LVEF network formed an open loop; therefore, a consistency model was employed.

DAPA (SMD = 0.34, 95% CI: –0.97 to 1.65) demonstrated the best performance among all treatment regimens, although the difference compared to SOC did not reach statistical significance. Furthermore, other treatment regimens did not show significant improvements in LVEF when compared with SOC or with each other, with no statistically significant differences observed. Details are provided in **Figures 3D**, **4B**.

#### 3.2.5 Safety and toxicity

The incidence of adverse reactions varied among the different treatment regimens, with statistically significant differences observed in the following comparisons: IPRA versus SEMA (OR = 0.26, 95% CI: 0.09–0.80), SEMA versus GLIM (OR = 0.46, 95% CI: 0.28–0.75), and GLIM versus SOC (OR = 0.67, 95% CI: 0.18–2.44). These findings indicate that IPRA is associated with a significantly lower incidence of adverse reactions compared to SEMA, while SEMA exhibits a significantly lower risk of adverse reactions compared to GLIM. Additionally, no statistically significant differences were observed between SOC and SOTA (OR = 0.49, 95% CI: 0.19–1.61) and between DAPA and EMPA (OR = 0.55, 95% CI: 0.06–5.16). No new safety concerns were identified in the included studies.

**Figure 5:**
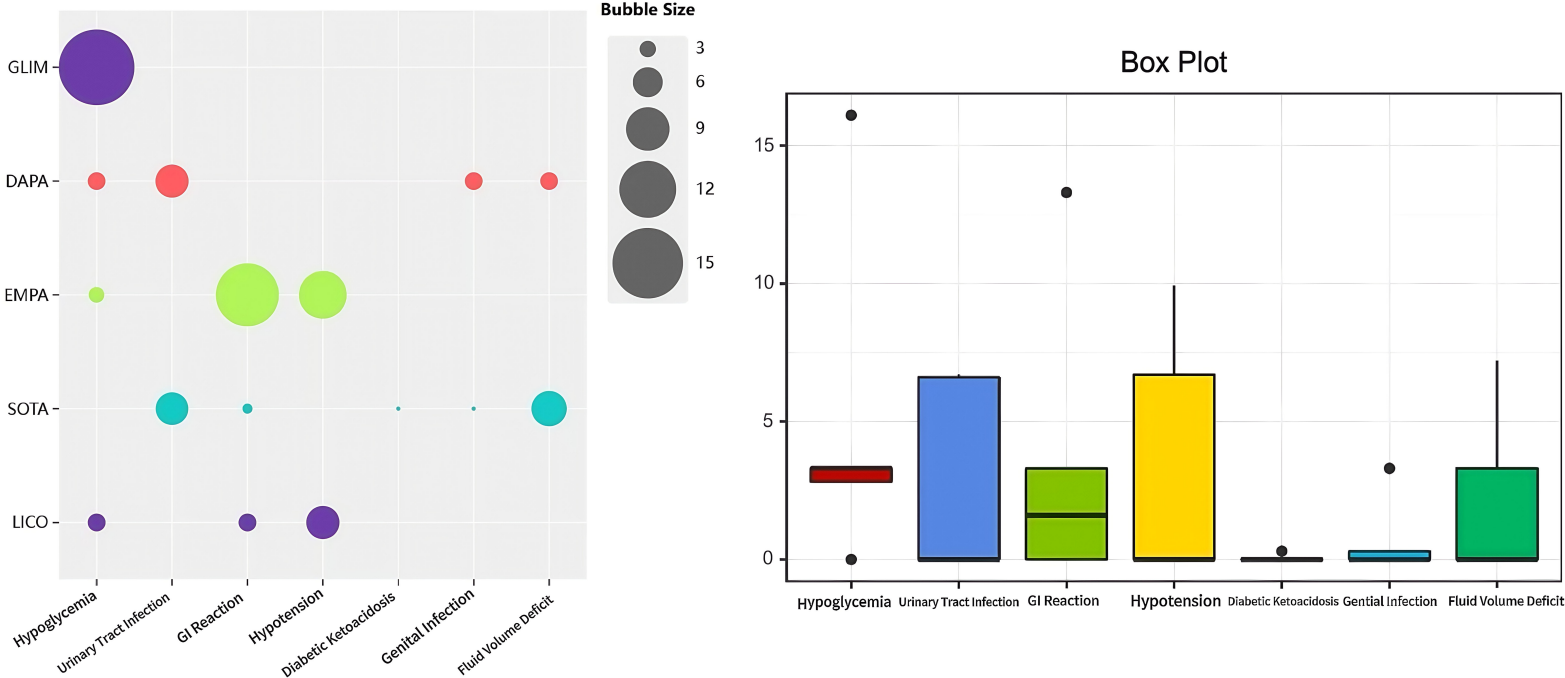
Safety profiles of various treatment regimens: Based on the results from the bubble plots and box plots, GLIM was found to be the most likely to induce hypoglycemia. DAPA and standard of therapy A (SOTA) were associated with an increased risk of urinary tract infections, whereas EMPA primarily manifested gastrointestinal reactions and a tendency towards hypotension. In contrast, LICO and IPRA exhibited relatively lower overall rates of adverse events, indicating a higher safety profile. However, the safety of DAPA and EMPA still requires further attention.

Common adverse events included hypoglycemia, urinary tract infections, gastrointestinal reactions, and hypotension (**Figure 5**). Details are provided in **Figure 4C**.

### 3.4 Rank analysis

#### 3.4.1 BNP outcome

**Figure 6:**
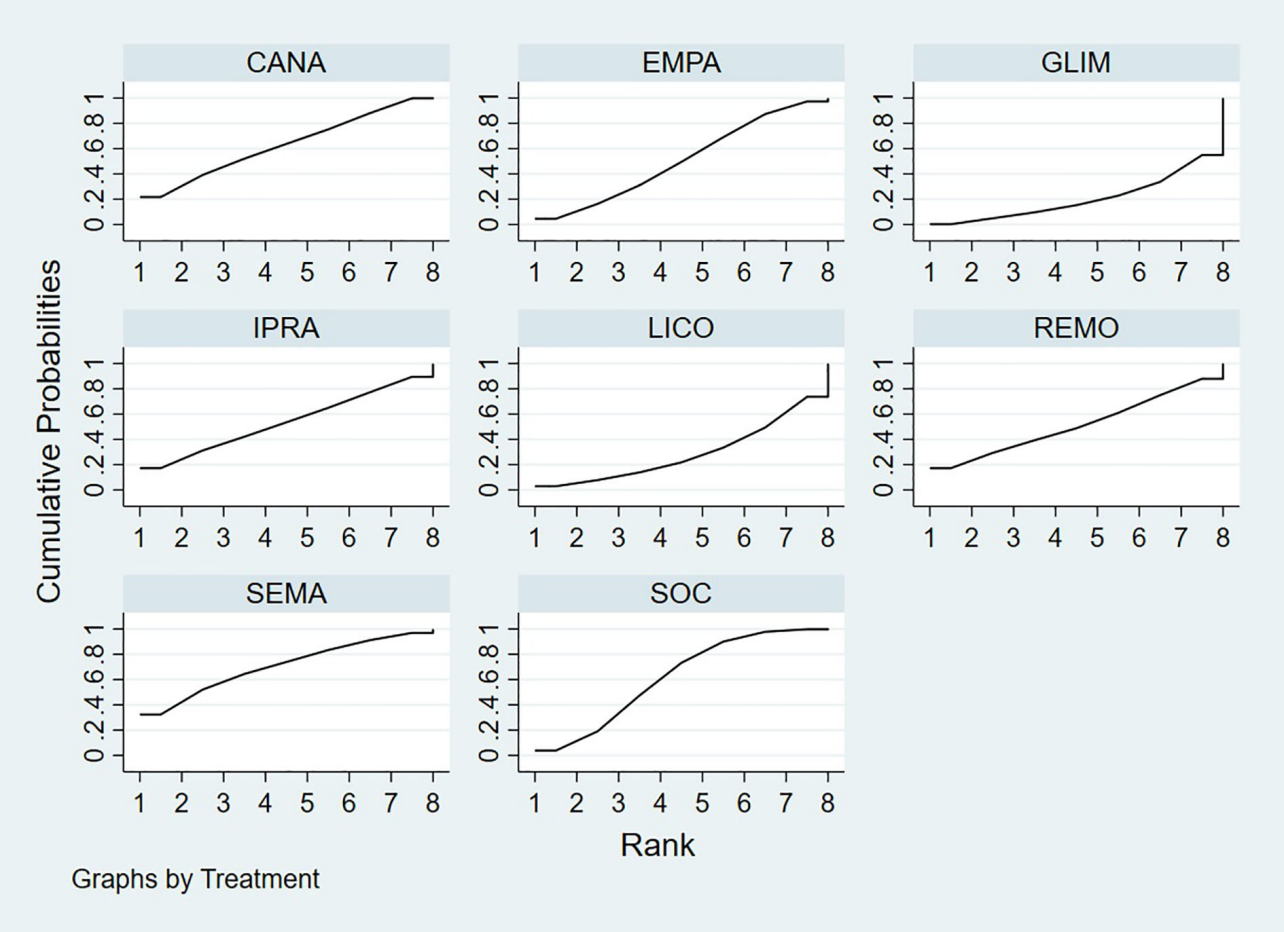
Bayesian rankogram illustrating the efficacy of different medications on BNP levels in patients.

Bayesian ranking analysis (**Figure 6**) revealed that SEMA is most likely to achieve the highest rank in improving BNP levels, with a cumulative probability of 31.3%, thereby demonstrating superior performance among the evaluated treatments. CANA follows with a 22.9% probability of attaining the top rank, while IPRA ranks third at 17.5%. LICO exhibits lower efficacy in BNP improvement, with only a 3.2% likelihood of securing the highest position. In contrast, GLIM performs the poorest, showing a 46.1% probability of being ranked last among all treatment options, indicating the least favorable outcome in BNP.

#### 3.4.2 Body weight outcome

**Figure 7:**
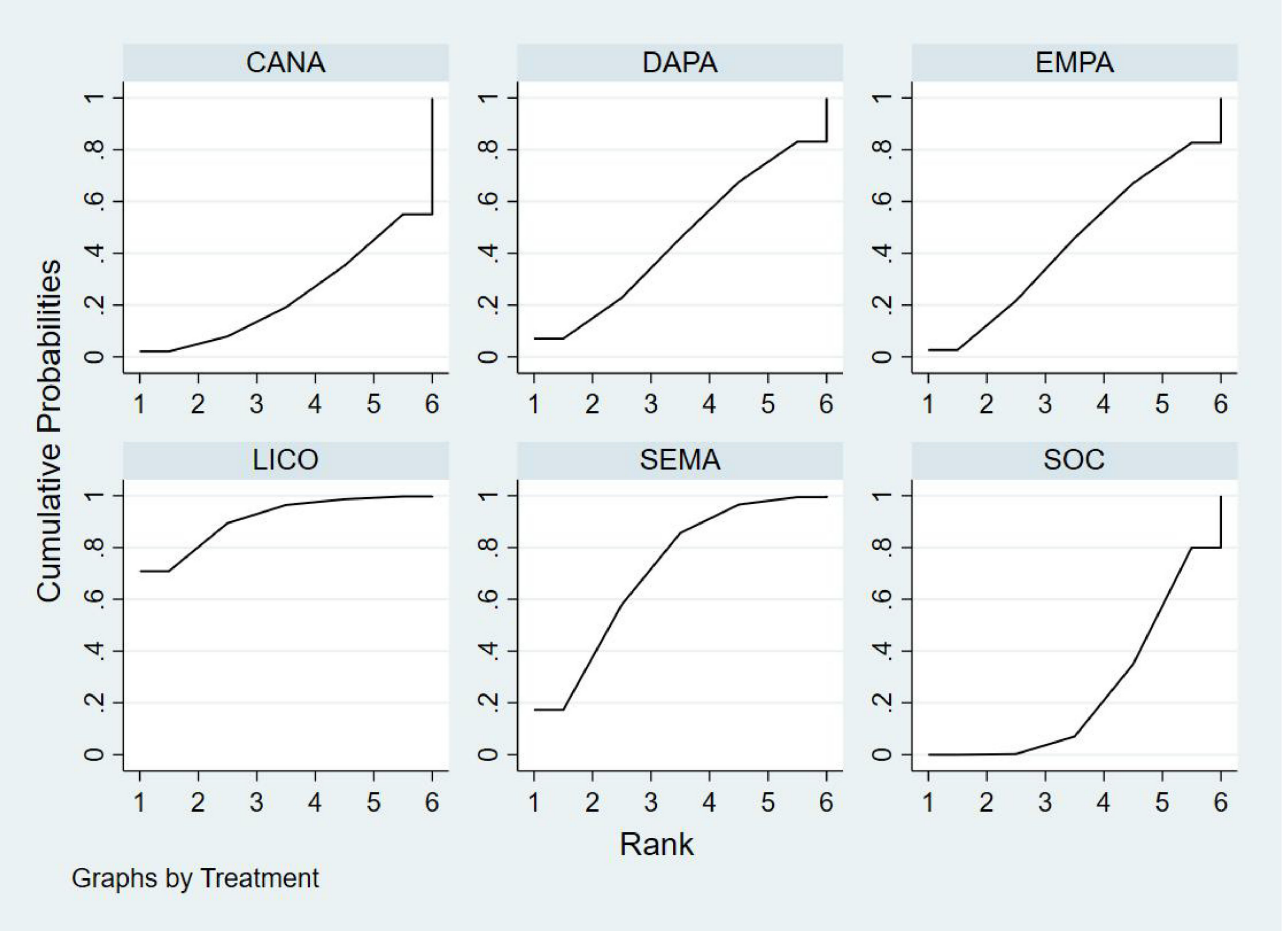
Bayesian rankogram illustrating the efficacy of different medications on body weight outcomes in patients.

Bayesian ranking analysis (**Figure 7**) revealed that LICO is most likely to achieve the highest rank in improving body weight, with a cumulative probability of 70.9%, demonstrating the most significant effect among the evaluated treatments. SEMA follows, ranking second with a 17.3% probability of attaining the top position. DAPA (7.0%), EMPA (2.7%), and CANA (2.1%) exhibit lower probabilities of securing the first rank, while SOC shows no likelihood of ranking first (0.0%).

In terms of the worst rankings, CANA performs the least favorably, with a 44.9% probability of being ranked last. In contrast, LICO and SEMA have minimal probabilities of occupying the lowest ranks, at only 0.3% and 0.6%, respectively. These findings further underscore the favorable efficacy and safety profiles of LICO and SEMA in managing body weight among patients with T2DM and HF.

#### 3.4.3 Glycated Hemoglobin outcome

**Figure 8:**
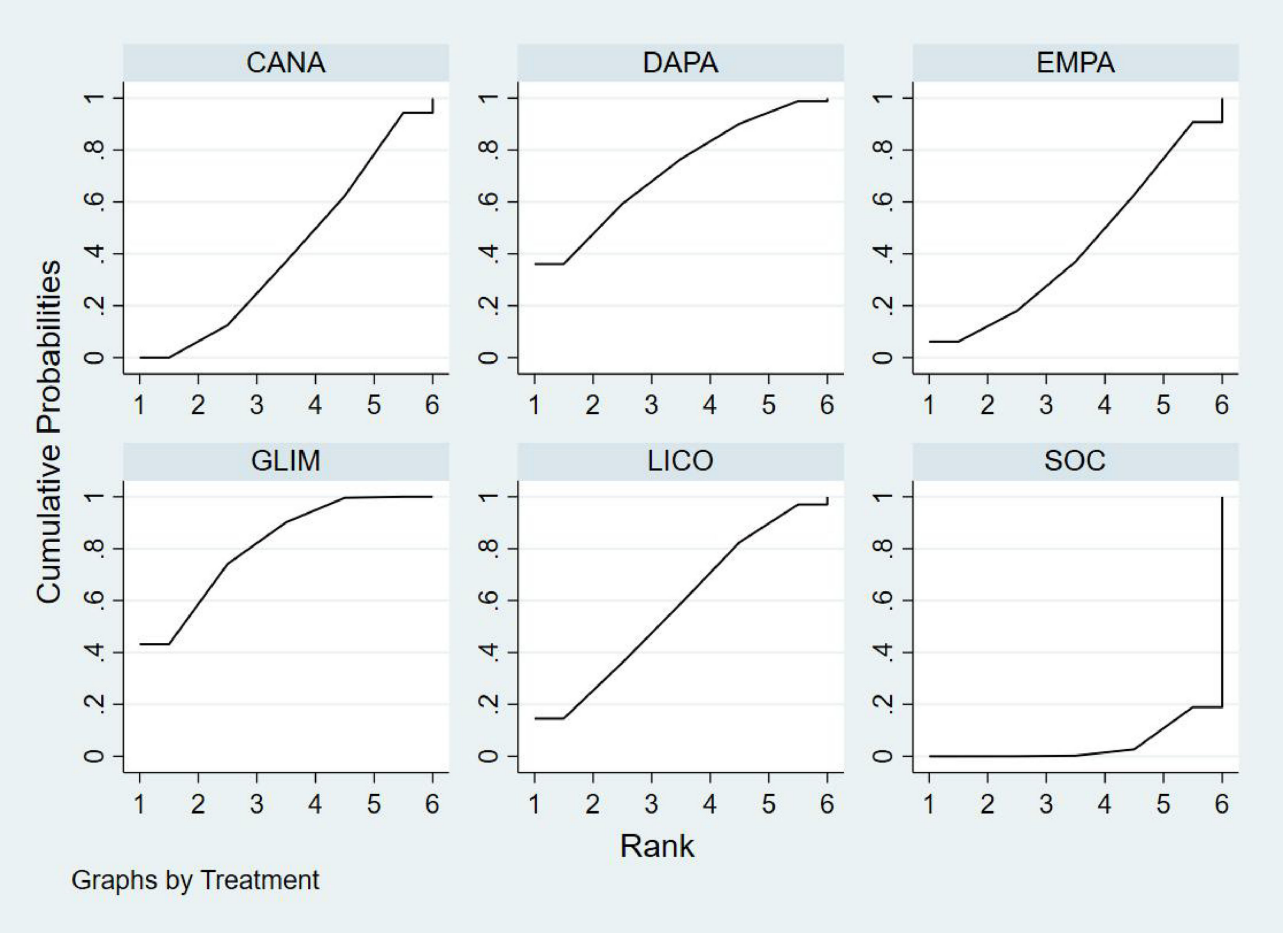
Bayesian rankogram illustrating the efficacy of different medications on HbA1c outcomes in patients.

Bayesian ranking analysis (**Figure 8**) indicated that GLIM is most likely to achieve the highest rank in improving HbA1c levels, with a cumulative probability of 43.2%, thereby demonstrating the best performance among the evaluated treatments. DAPA closely follows, with a 36.1% probability of attaining the top rank, while LICO ranks third with a 14.5% probability. CANA and SOC were the least effective in improving HbA1c, each exhibiting a 0.0% probability of securing the first rank.

Regarding the worst rankings, SOC displayed the most unfavorable performance, with an 81.1% probability of being ranked last. In contrast, GLIM showed no likelihood of being the worst performer (0.0%), underscoring its consistent efficacy in improving HbA1c levels.

#### 3.4.4 Left ventricular ejection fraction outcome

**Figure 9:**
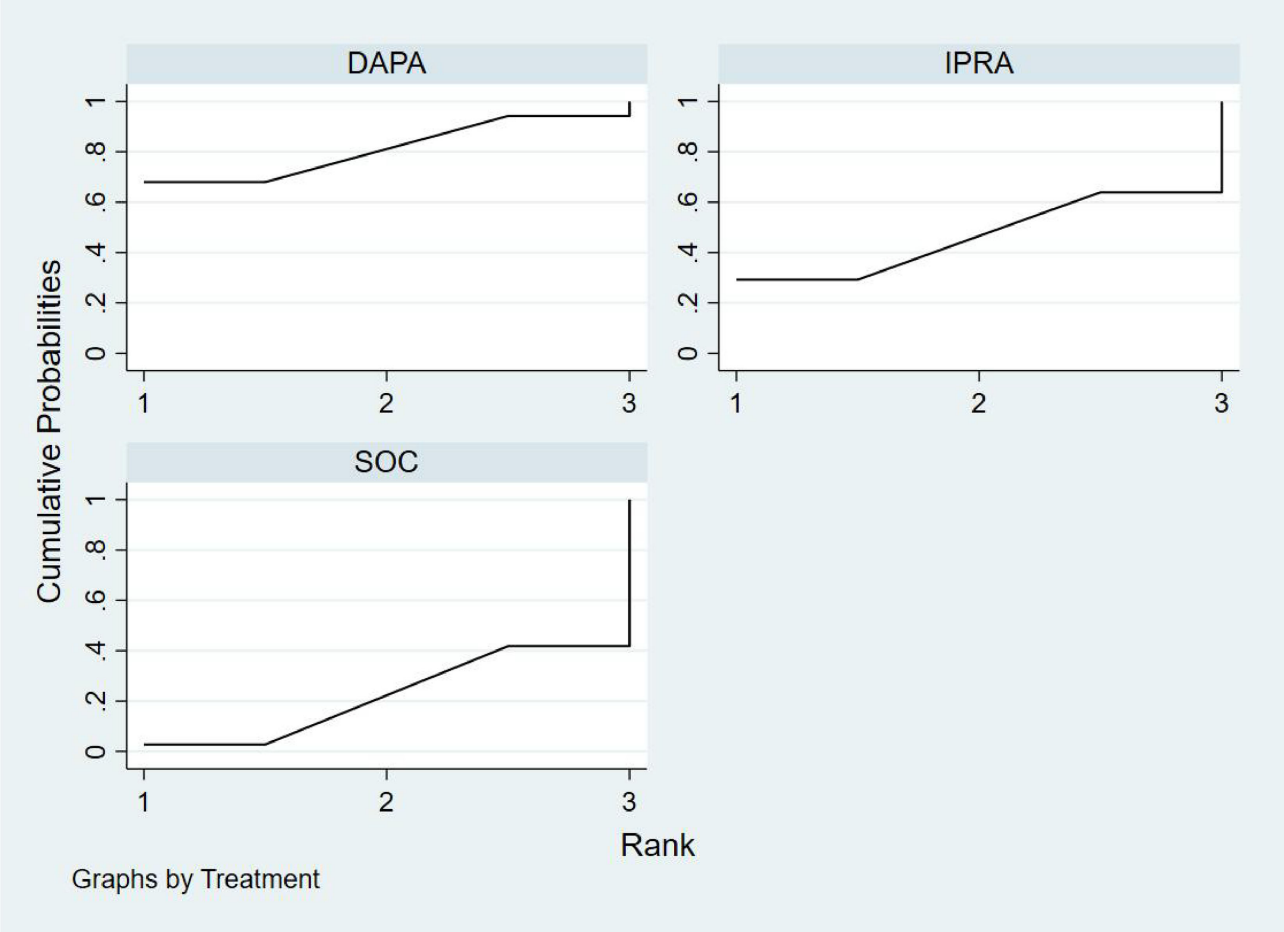
Bayesian rankogram illustrating the efficacy of different medications on LVEF outcomes in patients.

Bayesian ranking analysis (**Figure 9**) revealed that DAPA demonstrated the most significant improvement in LVEF, achieving the highest probability of ranking first with a cumulative probability of 68.0%. IPRA ranked second, possessing a 29.2% probability of attaining the top position. In contrast, SOC exhibited the poorest performance, with only a 2.8% probability of ranking first and a substantial 58.2% probability of being ranked last. These findings highlight SOC’s inferior efficacy in enhancing LVEF compared to the other treatment options.

#### 3.4.5 Adverse event incidence analysis

**Figure 10:**
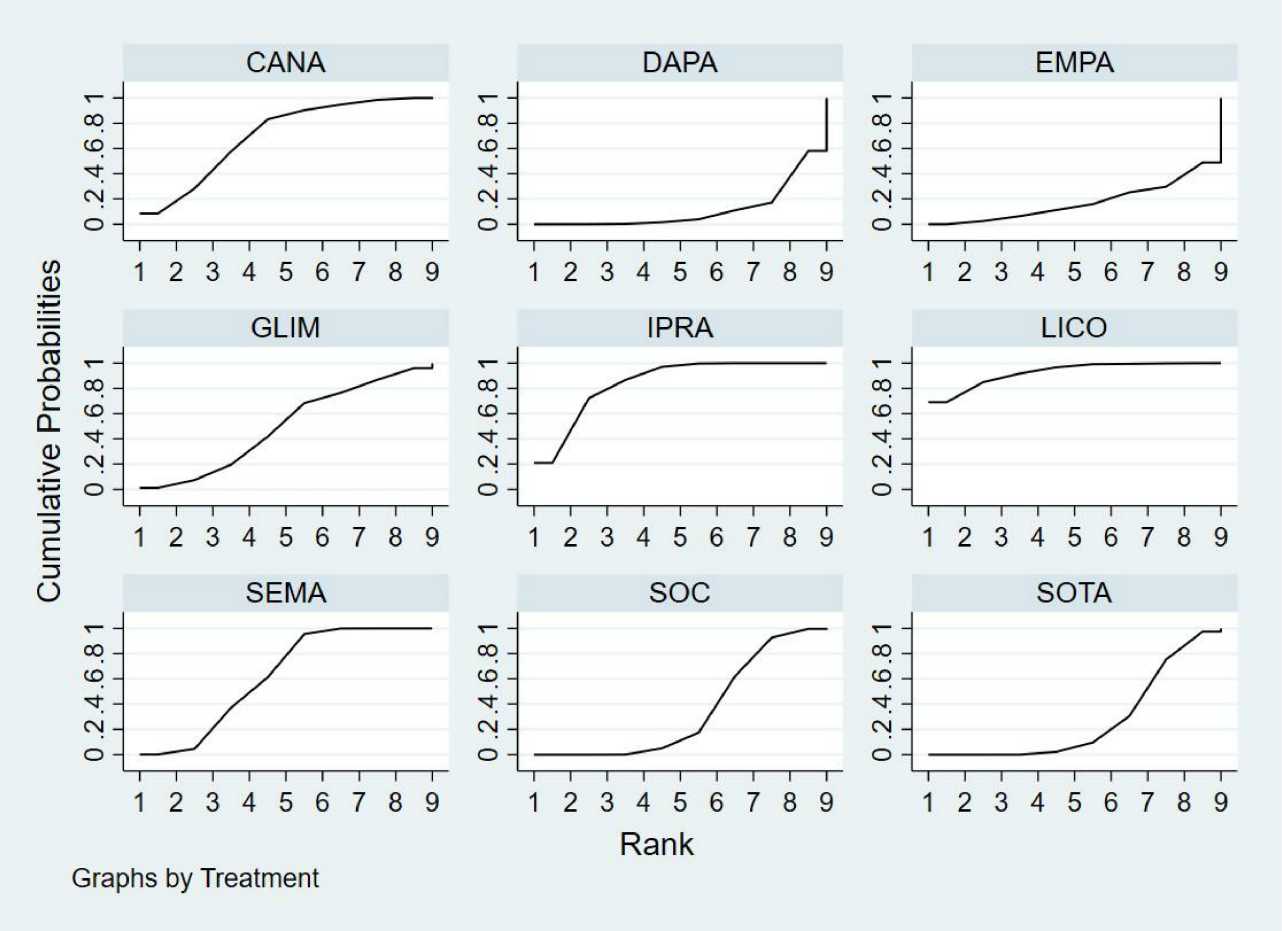
Bayesian rankogram illustrating the safety of different medications used in patients.

Bayesian ranking analysis (**Figure 10**) demonstrated that LICO exhibited the highest efficacy in controlling adverse events, with a 69.1% probability of ranking first. This indicates an exceptionally low risk of adverse event occurrence for LICO. IPRA followed with a 20.9% probability of attaining the top rank. In contrast, DAPA (41.9%) and EMPA (51.2%) showed higher probabilities of being ranked worst, reflecting a greater incidence of adverse events. Additionally, SOC and SOTA were present in the lower rankings, with SOC at 0.4% and SOTA at 2.6%. However, their performance was relatively better compared to DAPA and EMPA.

#### 3.4.6 Publication bias

**Figure 11:**
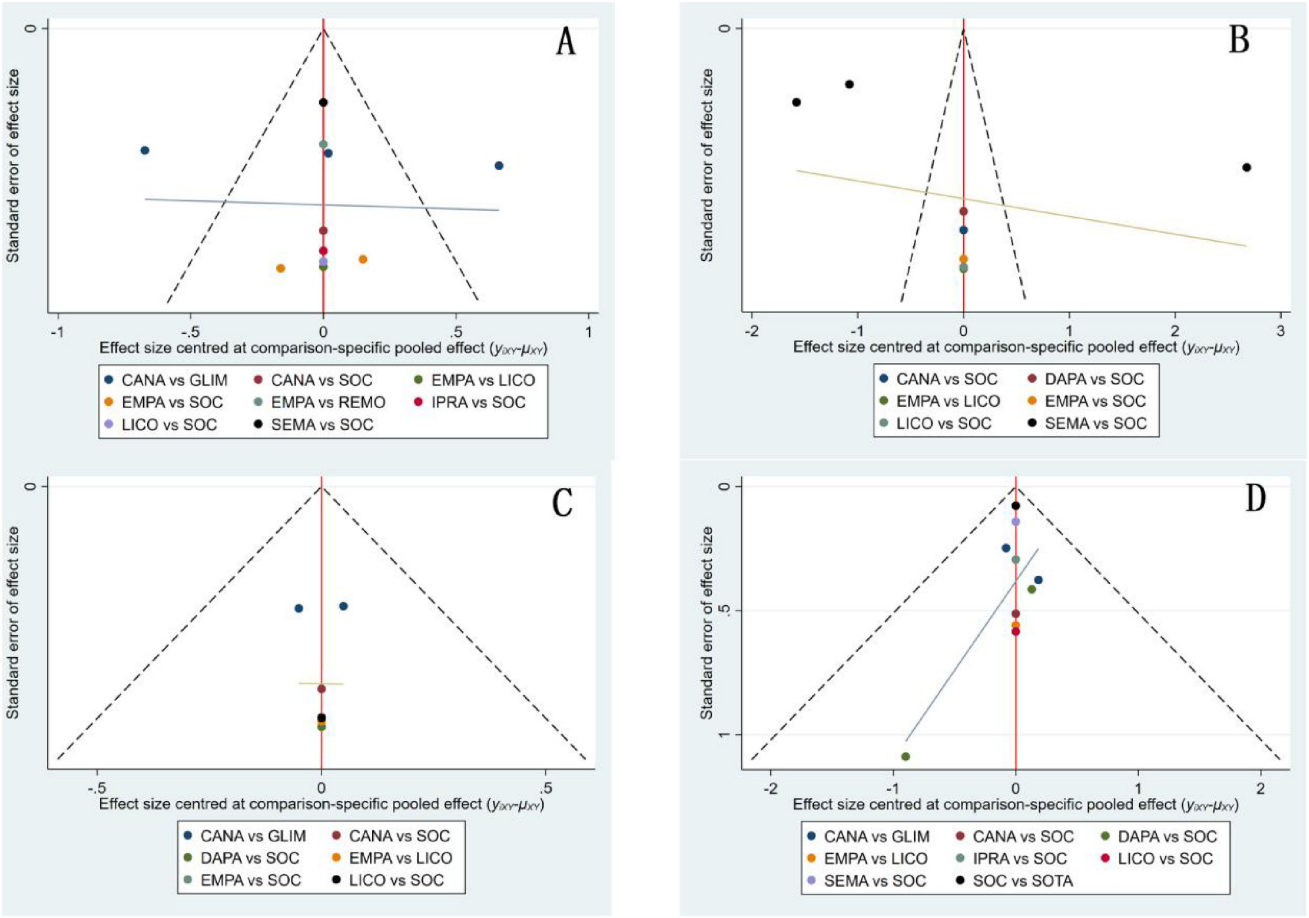
Funnel plots for patients using various medications. **(A)** BNP funnel plot; **(B)** Body weight funnel plot; **(C)** HbA1c funnel plot; **(D)** Drug safety funnel plot.

Funnel plots were generated for BNP, body weight, HbA1c, and drug safety outcomes to assess the presence of publication bias(**Figure 11**). The results demonstrated a symmetrical distribution of study points without any scattered outliers, indicating a minimal likelihood of publication bias in this study.

## 4 Discussion

This study is the first to systematically integrate three distinct classes of drugs—SGLT2 inhibitors, GLP-1 receptor agonists, and sulfonylureas—and compare their efficacy and safety in patients with T2DM and heart failure through a network meta-analysis. Unlike previous randomized controlled trials or traditional meta-analyses, our research consolidates multiple direct and indirect sources of evidence, providing a comprehensive therapeutic evaluation for this complex patient population. The results suggest that SEMA may be associated with improvements in BNP levels and body weight; however, these findings were not consistently statistically significant across comparisons, and should be interpreted as indicative trends rather than definitive effects. This suggests potential utility for patients needing both metabolic and cardiac support, but additional comparative trials are warranted to establish clinical superiority. Our findings are consistent with the meta-analysis by Barbagelata, which also confirmed the positive effects of SEMA on BNP reduction and weight improvement (25). However, this analysis included only six randomized controlled trials (RCTs), primarily comparing SEMA with placebo, and did not encompass drugs with other mechanisms of action. Additionally, Kristensen’s meta-analysis further supports that semaglutide significantly reduces BNP levels and body weight (26). However, this analysis included only six RCTs, primarily comparing SEMA with placebo, and did not encompass drugs with other mechanisms of action. Additionally, Kristensen’s meta-analysis further supports that semaglutide significantly reduces BNP levels and body weight. By incorporating additional drug classes such as SGLT2 inhibitors and sulfonylureas, our analysis extends the comparative framework and validates SEMA’s effects across a broader therapeutic landscape. It further validated the advantages of SEMA across multiple outcomes, addressing the limitations of existing research.

LICO ranked first in body weight improvement, demonstrating its unique role in metabolic control, particularly suitable for patients with T2DM and heart failure who are also obese. Our findings are consistent with Cheong’s meta-analysis, which confirmed the significant effect of LICO in weight management and highlighted the potential advantages of combined SGLT1/SGLT2 inhibition in reducing body weight (27). However, this study did not directly compare LICO with GLP-1 receptor agonists or other medications that operate through different mechanisms of action.

Furthermore, the results of this study are consistent with those of Teo’s meta-analysis, which also demonstrated a significant effect of LICO in weight management. However, Teo’s study primarily focused on comparisons with placebo or other single SGLT2 inhibitors, without including medications with different mechanisms of action (28). This limitation is addressed in our study by incorporating a broader range of medications and more comprehensive outcome measures, thereby expanding the potential application value of LICO in weight management and metabolic improvement. Finally, Zaki’s meta-analysis further confirmed the dose-dependent effect of LICO in weight management, particularly at higher doses (150 mg once daily), where weight reduction was most significant (–4.20 kg) (29). However, the current study did not encompass a comprehensive comparison of LICO with other medications that operate through different mechanisms of action. In contrast, our study provides broader clinical evidence support through a multi-drug comparative analysis.

DAPA was associated with the highest probability of improving LVEF in Bayesian ranking analysis. However, However, the credible intervals crossed the null line, indicating no statistical significance; thus, this should be interpreted as a potential trend rather than a confirmed therapeutic advantage. These findings may reflect underlying hemodynamic and metabolic effects of DAPA, although further head-to-head trials are needed to confirm its superiority. Our findings are consistent with those of Jhund, whose analysis validated the efficacy of DAPA in enhancing cardiac function and metabolic parameters (30). However, their study was limited to comparisons between DAPA and placebo, without including other SGLT2 inhibitors or medications with different mechanisms of action. Additionally, Zannad’s meta-analysis highlighted that DAPA significantly improved LVEF in patients with HFrEF and reduced the risk of heart failure hospitalization (31). Nevertheless, their research did not cover a wider range of SGLT2 inhibitors nor involve direct comparisons with GLP-1 receptor agonists or other classes of medications. By incorporating multiple drugs and analyzing a broader spectrum of outcome measures, our study not only expands the research scope but also addresses the limitations of single-trial analyses, thereby providing more comprehensive evidence to support clinical decision-making.

GLIM exhibited moderate efficacy in glycemic control, consistent with its insulin-stimulating mechanism. However, it showed a higher likelihood of hypoglycemia and lacked additional cardiovascular benefits, limiting its suitability for heart failure patients. Its lower cost may still make it a practical option under economic considerations.

Interestingly, SEMA showed significant performance in metabolic improvement, contrasting sharply with its limited effect on cardiac function. This discrepancy may suggest different regulatory mechanisms between metabolic outcomes and cardiac function improvements, warranting further investigation in future studies.

The strengths of this study lie in the inclusion of multiple SGLT2 inhibitors (such as CANA, IPRA, EMPA, REMO, LICO, and DAPA), which comprehensively reflect the overall efficacy and safety profiles of this drug class, thereby avoiding the limitations associated with single-drug studies. Additionally, this study simultaneously evaluated three classes of drugs with different mechanisms of action—SGLT2 inhibitors, GLP-1 receptor agonists, and sulfonylureas—systematically analyzing their efficacy and safety. Compared to previous studies that focused on a single mechanism or a limited range of drugs, our research offers broader coverage, providing more comprehensive evidence-based guidance for clinical practice.

Nonetheless, this study has certain limitations. First, the included RCTs predominantly involved patients from Europe, North America, and Asia, limiting the generalizability of the findings to underrepresented populations, such as those from Africa or Latin America. Future studies should aim to include more diverse patient populations. Second, the study lacked data on patient-reported quality of life outcomes, thereby preventing a comprehensive assessment of the potential impact of these medications on patients’ quality of life. Furthermore, most trials had relatively short follow-up periods, which are inadequate to fully reveal the long-term efficacy and safety of the medications. In addition, due to the limited number of trials reporting heart failure subtypes, we were unable to analyze outcomes separately for patients with preserved versus reduced ejection fraction. Given the distinct pathophysiology and treatment responses of HFrEF and HFpEF, this represents an important area for future research. Addressing these gaps will require studies with longer follow-up durations to provide a more thorough understanding of the long-term effects.

## 5 Conclusion

This study compared the efficacy and safety of SGLT2 inhibitors, GLP-1 receptor agonists, and sulfonylureas in managing type 2 diabetes with heart failure. SEMA demonstrated a trend toward improving metabolic control and BNP levels, LICO ranked highest in weight reduction probability, while DAPA exhibited potential in enhancing both HbA1c and LVEF, though these results did not always reach statistical significance. LICO and IPRA were associated with more favorable safety profiles, while GLIM had a higher risk of hypoglycemia.

These findings provide a comparative overview that may assist clinicians in tailoring treatment strategies according to individual patient profiles and comorbidities.

However, further studies are needed to assess long-term outcomes, especially in underrepresented populations, and to determine the consistency of treatment effects across various heart failure phenotypes.
